# Physical Responses to the Structural Evolution of CL-20-Based Energetic Solvates with H_2_O_2_ and H_2_O Under External Electric Field

**DOI:** 10.3390/ijms27167209

**Published:** 2026-08-12

**Authors:** Lu Shi, Li Fan, Huaya Sun, Daichuan Ma

**Affiliations:** 1 College of Chemistry and Materials Engineering, Mianyang Teachers’ College, Mianyang 621000, China; 18280769523@163.com (L.F.); shy1738082@outlook.com (H.S.); 2 Analytical & Testing Center, Sichuan University, Chengdu 610064, China; dcma@scu.edu.cn

**Keywords:** energetic solvates, external electric field, structural evolution

## Abstract

The effects of an external electric field (EEF) on the crystal structure, nonbonded interaction, sensitivity, and mechanical properties of CL-20/H_2_O_2_ and CL-20/H_2_O energetic solvates have been investigated by classical molecular dynamics simulations. Compared with CL-20/H_2_O solvate, CL-20/H_2_O_2_ solvate possesses stronger O-H···O and C-H···O hydrogen-bond networks, which are more sensitive to the variation of electric field intensity. Such hydrogen-bond frameworks buffer the fluctuation of cell parameters and restrain molecular diffusion under an EEF. Increasing the electric field shortens the N-NO_2_ trigger bond and reduces its interaction energy, strengthening the intrinsic sensitivity of CL-20 solvates. The conformers of CL-20 molecules transform from the *α*-phase to the stable *ε* phase after 0.4 V·Å^−1^. When an external electric field acts on the CL-20/H_2_O_2_ and CL-20/H_2_O energetic solvates, conformational changes in CL-20 molecules and solvation interactions can effectively reduce stiffness and enhance lattice ductility, reducing hotspot formation and thereby improving thermal safety.

## 1. Introduction

Energetic materials, as a special type of fine chemical product, are widely used in gunpowder, explosives, propellants, and pyrotechnics, playing an important role in national defense and the national economy [1,2,3]. Their high nitrogen content and outstanding thermal stability make them have significant application potential in fields such as explosives and propellants. The study of the effects of external electric fields (EEFs) on the properties of energetic materials provides important guidance for designing and synthesizing new explosives that combine safety, thermal stability, and high-energy insensitivity. 2,4,6,8,10,12-hexanitro-2,4,6,8,10,12-hexacyclohexadecane (CL-20) is a currently outstanding cage-type nitramine explosive with excellent comprehensive performance, high energy density and detonation performance, whereas its application was limited by the high mechanical sensitivity. As a novel modification technology, energetic materials combine two or more different molecules through non-covalent bonds into the same lattice, forming a multi-component crystal with a specific structure and properties, such as reducing the sensitivity of explosives and improving the density, oxygen balance and thermal stability of explosives [4,5].

To reduce the mechanical sensitivity, some insensitive energetic materials were selected as ligands to form eutectics with CL-20, effectively enhancing their density, thermal stability, and mechanical sensitivity [6,7,8]. In recent years, CL-20 has formed cocrystals with various compounds (both energetic and non-energetic), including the early CL-20/TNT and CL-20/HMX energetic cocrystals [9,10]. The Nitro-π interaction was performed on the electron-deficient ring of BTF in CL-20/BTF cocrystals, and its detonation performance was superior to that of BTF [11]. In 2016, the polymorphic form of CL-20/hydrogen peroxide (HP) energetic solvates (orthorhombic and monoclinic) were formed with a higher crystal density (respectively 2.03 and 1.96 g/cm^3^) and higher detonation velocity and pressure, approaching the level of ε-CL-20, effectively improving the oxygen balance and thermal stability of CL-20 explosives [12]. Two new DNBT/hydrogen peroxide (HP) solvates are also similar to the above CL-20-based multi-component materials [13]. These materials employ multiple bonding modes with CL-20 and emphasize the importance of an in-depth understanding of weak intermolecular interactions, such as C-H···nitro, for the design of multi-component energetic substances, thereby confirming the potential of CL-20 as a universal cocrystal former.

The influence of temperature and pressure on the structure and properties of explosives has been comprehensively studied through theory and experiments [14,15,16,17,18,19]. However, theoretical research on energetic solids under external electric fields has not yet been carried out, although accidents caused by static electricity in explosive production plants easily occur, which is partly attributed to the fact that the influence of external electric fields on the structure and properties of energetic materials is still not fully understood. The effect of external electric fields on the sensitivity of explosives is analyzed by the molecular and electronic structure, the frontier molecular orbital energy gap, AIM electron density analysis and the electrostatic potential [20,21,22]. In 2015, the effect of external electric fields on the decomposition rate of crystalline lead azide using periodic first-principles calculations was explored [23]. The effect of external electric fields on the influence of energetic pentazole crystals on the energetic cocrystals at the molecular level, through parameters such as bond dissociation energy of N-NO_2_, frontier molecular orbitals, electrostatic potential (ESP), and nitro group charge (Q_NO2_), has also been studied [24,25]. Furthermore, the buffering mechanism of external electric fields on the initial thermal decomposition process of a series of chain-catenated Nx (x = 4, 8, 10) energetic crystals and energetic pentazole crystals has been elucidated using first-principles calculations [26,27]. In addition, Politzer et al. studied the effect of external electric fields on five typical trigger bonds, clarifying the type of bond breakage that plays a key role in detonation initiation [28]. The transfer of electrons and oxygen atoms in nitro groups is crucial in energetic material triggering reactions, and N-NO_2_ is usually the trigger bond of nitramine explosives [29]. The influence of external electric fields on the bond dissociation energy and energy barrier of the trigger bonds of C-NO_2_ or N-NO_2_ bond in CH_3_NO_2_ or NH_2_NO_2_ and BITE-101 crystals was systematically studied [30,31]. However, studies on the sensitivity and other performance of CL-20-based energetic solvate cocrystals under electric fields are relatively rare, which hinders a deeper understanding of the decomposition mechanism of energetic materials under external electric fields.

In this work, the structural evolution and physical response of CL-20/H_2_O_2_ and CL-20/H_2_O energetic solvates were studied under an EEF applied along the *z*-axis using molecular dynamics (MD) simulations, with intensities ranging from 0.0 to 1.4 V·Å^−1^. Specifically, we analyze the gradient changes of crystal structure induced by an EEF, characterize the evolution of hydrogen bond networks via radial distribution functions, evaluate molecular mobility using root mean square displacement, explore the structural variations of the trigger bond (N-NO_2_), and quantify the anisotropic changes in mechanical properties and cohesive energy density of the solvates. The primary objectives of this study are to reveal the regulatory mechanism of an EEF on the structure-property relationships of CL-20-based solvates, clarify the differences in EEF response sensitivity between H_2_O and H_2_O_2_ solvent systems, and provide theoretical references for the safety optimization and performance tailoring of EMs under extreme electromagnetic environments. This theoretical research contributes to a deeper understanding of the decomposition mechanisms of energetic materials under external electric fields and has significant implications for their use and storage in complex electromagnetic environments.

## 2. Results and Discussion

### 2.1. Crystal Structures and Conformers

**Figure 1:**
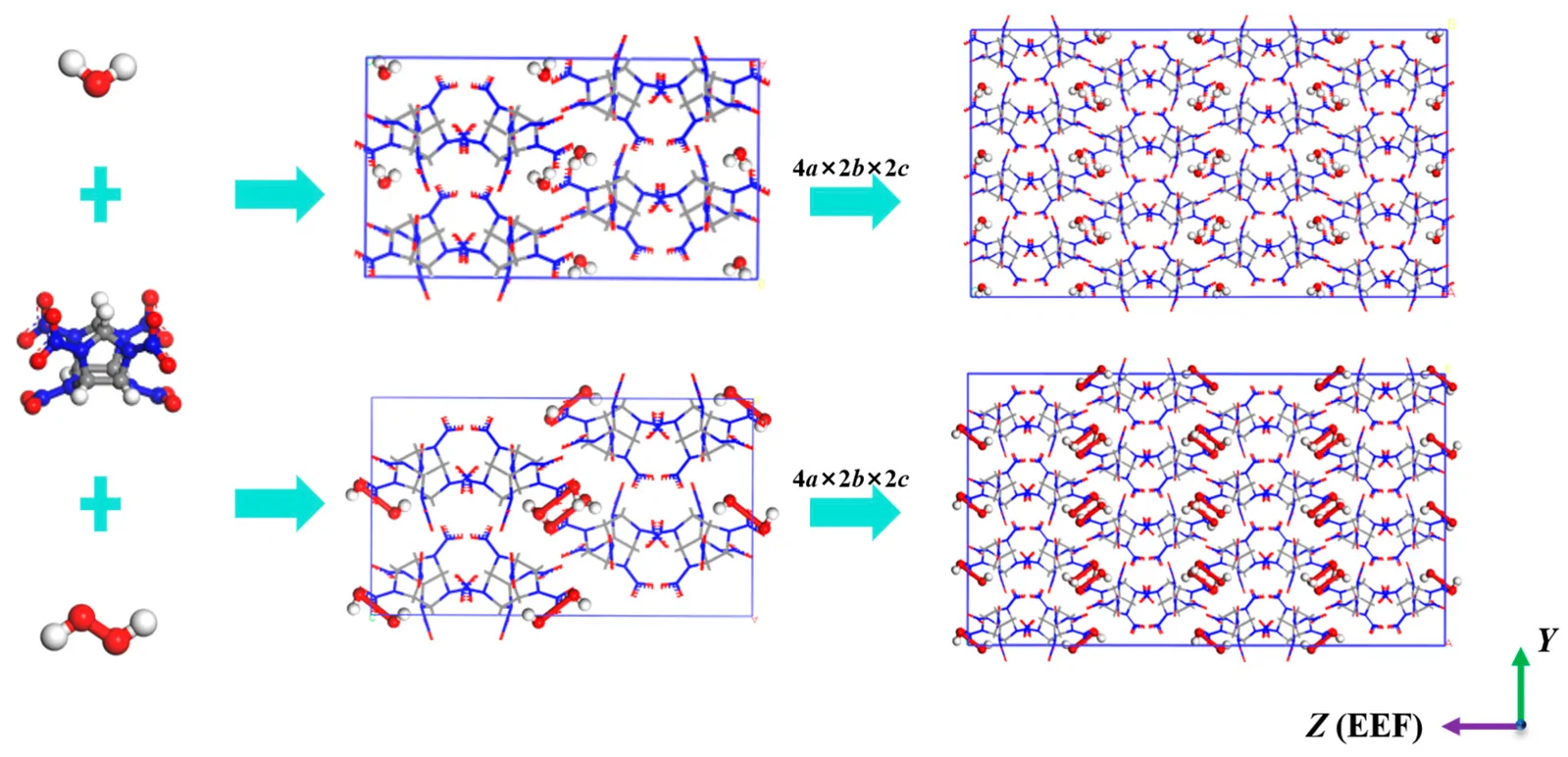
Unit cells and supercells of CL-20/H_2_O_2_ and CL-20/H_2_O energetic solvates. The crystal orientation relative to the applied electric field (*z*-axis) is indicated at the bottom-right corner.

To explore the effect of external electric fields on the conformational transformation of energetic solvate crystals, geometric relaxation of supercells was performed under different electric field intensities. All initial crystal configurations were derived from experimental low-temperature XRD data from the CCDC database and fully relaxed under ambient temperature and pressure, ensuring that all simulation models correspond to thermodynamically stable and experimentally conforming structures. The crystal structures of CL-20/H_2_O_2_ and CL-20/H_2_O solvates are shown in Figure 1. The electric field is applied along the positive crystallographic *z*-axis of optimized supercells with MD simulations, which is parallel to the orientation of N-NO_2_ trigger bonds of CL-20 inside the crystal lattice.

**Figure 2:**
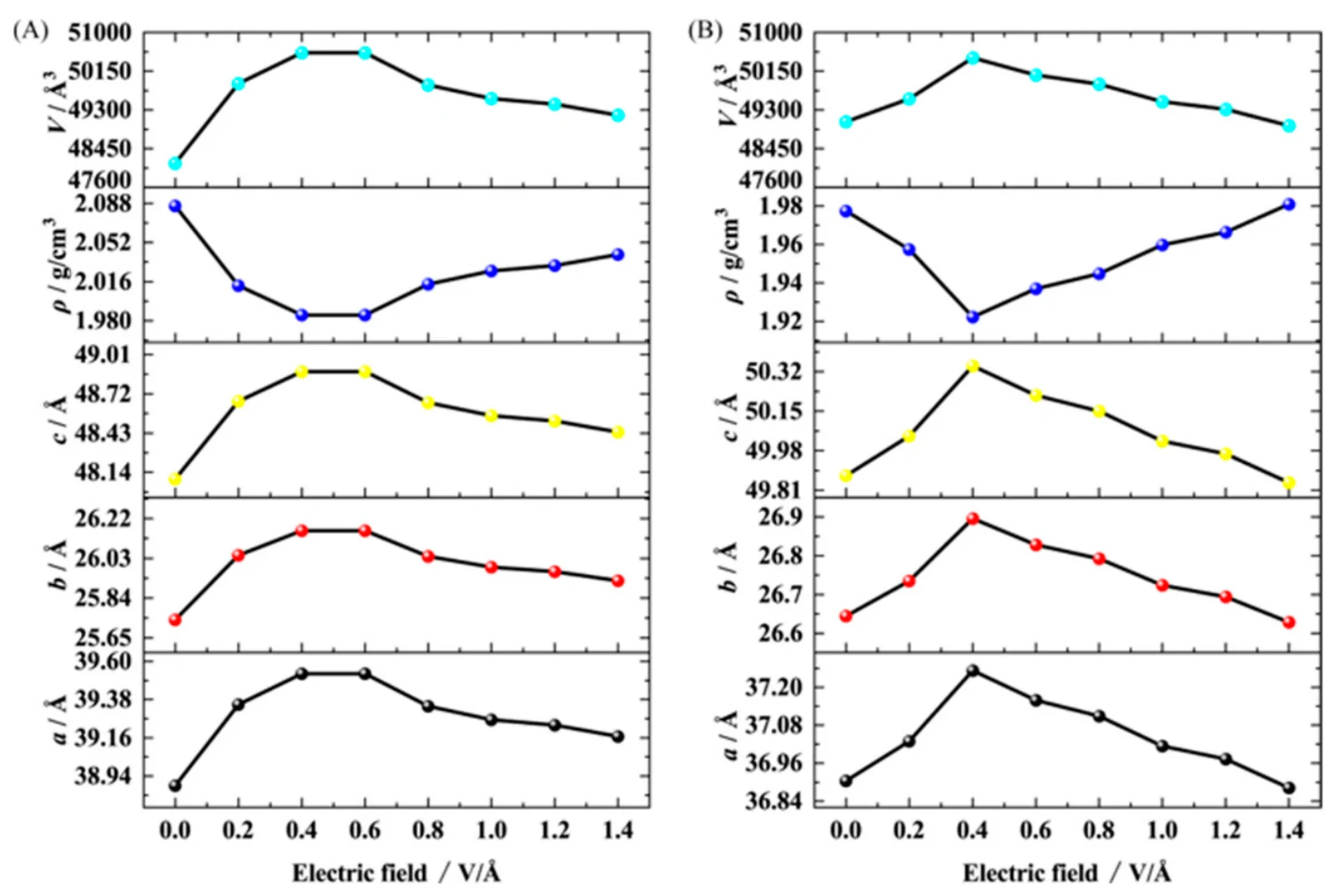
The variation for lattice parameters, volume, and density of (**A**) CL-20/H_2_O_2_ and (**B**) CL-20/H_2_O solvates under increased external electric fields applied along the *z*-axis.

Figure 2 shows the variations in lattice parameters, unit-cell volume, and density of the CL-20/H_2_O_2_ and CL-20/H_2_O solvates as a function of the applied electric field. For CL-20/H_2_O_2_, the lattice parameters a, b, and c initially increase with the field strength rising from 0.0 to 0.4 V/Å, plateau at 0.4–0.6 V/Å, and then decrease gradually. The unit-cell volume V also rises rapidly to a maximum in this range. As the field increases from 0.6 to 1.4 V/Å, the lattice parameters and volume decrease gradually, while the density increases slowly.

In contrast, the lattice parameters and density of CL-20/H_2_O (α-CL-20) first increase monotonically and then decrease over 0.0–1.4 V/Å. The amplitude of variation is significantly larger, indicating that the structure of CL-20/H_2_O is more sensitive to electric-field-induced polarization. This difference can be attributed to their distinct stoichiometries and crystal structures. CL-20/H_2_O_2_ crystallizes in the *P*_bca_ space group with a 2:1 stoichiometry and eight CL-20 molecules per unit cell, while CL-20/H_2_O has a 4:1 stoichiometry with two solvent molecules in the unit cell. The higher solvent content in CL-20/H_2_O_2_ enhances intermolecular interactions, stabilizes the crystal lattice, and buffers the structural deformation under external electric fields.

**Figure 3:**
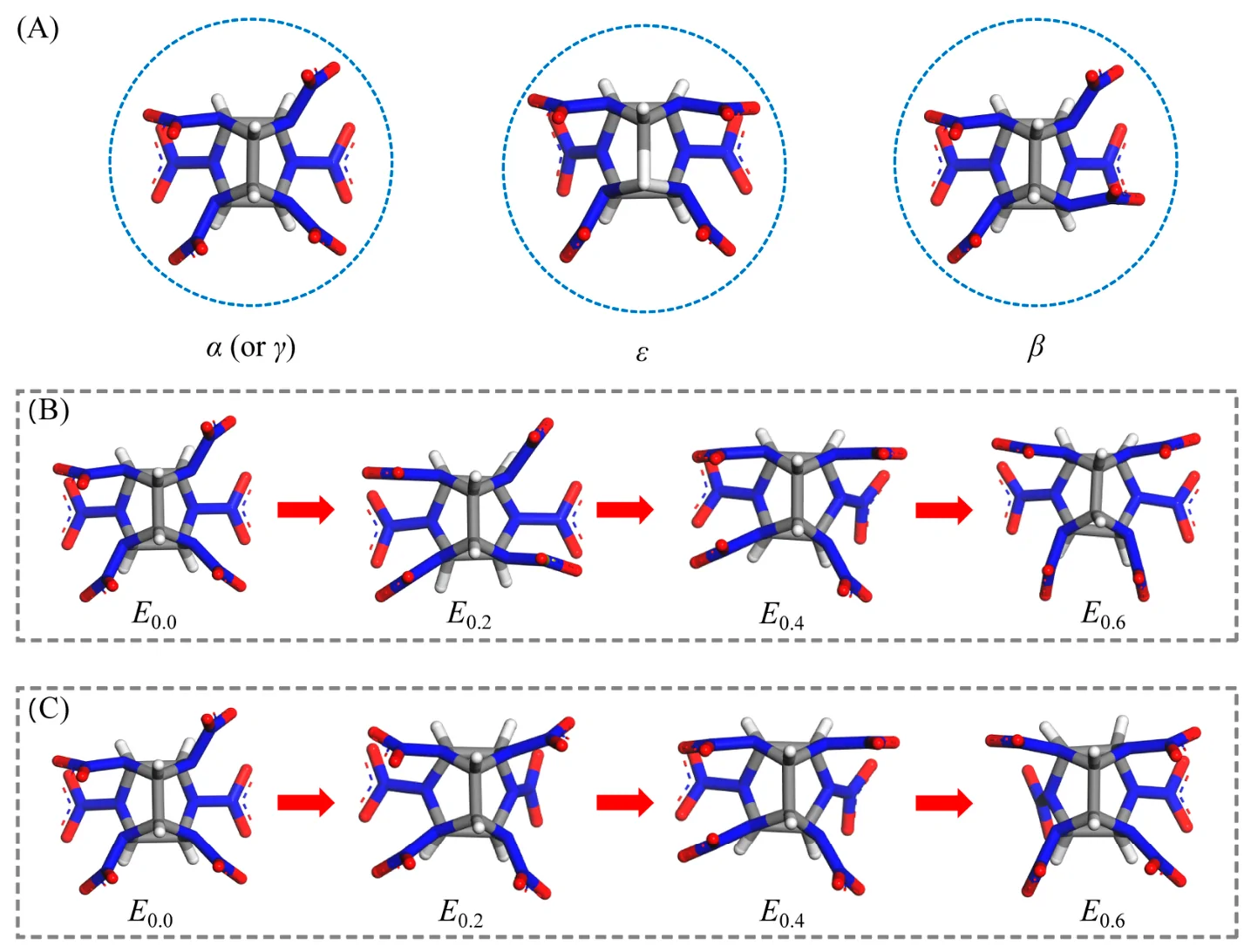
(**A**) The conformers of CL-20 molecules. (**B**) The conformer evolution of CL-20/H_2_O_2_ and (**C**) CL-20/H_2_O solvates under different intensities of external electric fields.

The difference in lattice stability mentioned above is essentially determined by the type of solvent guest molecules. CL-20-based energetic solvates were formed by substituting H_2_O molecules in the CL-20/H_2_O solvate (α-CL-20) with hydrogen peroxide. This breakthrough enables the cocrystallisation of small oxygen-containing molecules without sacrificing the energy level of the explosives. The CL-20 at different temperatures and pressures has been investigated with α, β, ε, and γ based on the difference in the initial orientation of the nitro group in the molecule and lattice packing, whose stability order is ε > γ > α > β [32,33,34,35]. The molecular structures of the four CL-20 polymorphs are shown in Figure 3A. Figure 3B,C show the structural evolution of CL-20/H_2_O_2_ and CL-20/H_2_O solvates under varying intensities of the external electric field applied along the *z*-axis. The results reveal that the space orientation of the NO_2_ groups of CL-20 in CL-20/H_2_O_2_ and CL-20/H_2_O solvates changes with increasing field strength and transforms into the stable ε conformers after 0.4 V·Å^−1^. It is due to changes in the orientation of the CL-20 molecular dipole moment induced by electric-field interactions between CL-20 molecules, as well as alterations in the chemical environment from solvent effects, which in turn lead to conformational changes.

### 2.2. Intermolecular Interactions Analysis

**Figure 4:**
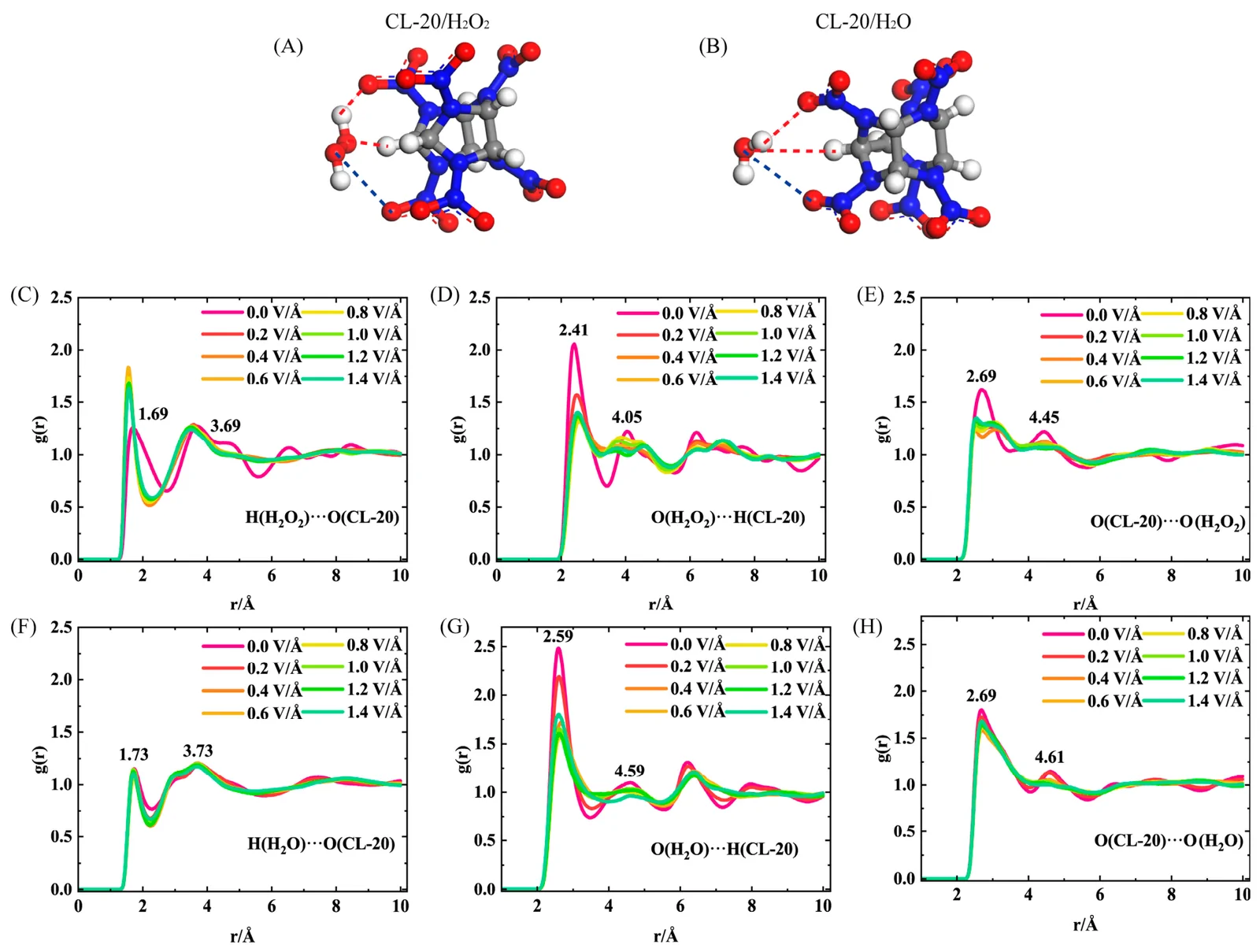
The extracted interaction pairs of O···H or O···O atoms (**A**,**B**). Radial distribution functions (RDFs) of special atom pairs in CL-20/H_2_O_2_ (**C**–**E**) and CL-20/H_2_O solvates (**F**–**H**).

Generally, radial distribution function (RDF) analysis can be used to measure local spatial ordering by providing the probability density g(*r*) of finding an atom at a distance *r* from a reference atom. Generally, the interaction distance range (*r*) for the hydrogen bond is 2.0–3.1 Å, and for strong van der Waals, it is 3.1–5.0 Å. The van der Waals (vdW) interactions become negligible beyond 5.0 Å [5]. Figure 4 shows the RDFs of O···H or O···O atomic pairs in CL-20/H_2_O_2_ and CL-20/H_2_O solvates under the increased electric field intensities. Here, two typical intermolecular hydrogen-bond configurations are defined as H (H_2_O_2_ or H_2_O)···O(CL-20) (solvent as H-donor, CL-20 nitro oxygen as H-acceptor) and O (H_2_O_2_ or H_2_O)···H(CL-20) (solvent oxygen as H-acceptor, CL-20 C-H as H-donor). The O···O contact between CL-20 and solvent molecules originates from intermolecular dipole electrostatic attraction between neutral molecules. No ionic dissociation of hydrogen or oxygen atoms is observed under external electric field. In Figure 4, the first intense peaks of the RDFs for H···O/O···H atom pairs in CL-20/H_2_O_2_ are located at 1.69 and 2.41 Å, which are shorter than the corresponding first peaks of H···O/O···H atoms pairs in CL-20/H_2_O (1.73 and 2.59 Å), respectively. In addition, the primary O···O atom pair peaks of both CL-20/H_2_O_2_ and CL-20/H_2_O solvates appear at 2.69 Å. These results reveal that intermolecular hydrogen bonding serves as the dominant interaction between CL-20 and solvent molecules, accompanied by van der Waals interactions. Moreover, the hydrogen-bonding interaction between CL-20 and H_2_O_2_ is stronger than that between CL-20 and H_2_O, where H_2_O_2_ molecules provide hydrogen bond donors.

In addition, based on the QTAIM (Quantum Theory of Atoms in Molecules) [36,37], the more detailed information about the hydrogen-bonding interactions can be obtained by conducting topological analyses on the molecular clusters extracted from the CL-20/H_2_O_2_ and CL-20/H_2_O solvates (seen from Figure S2). Table S1 present the topological parameters of the hydrogen bonds in the CL-20/H_2_O_2_ and CL-20/H_2_O solvates, respectively. The electron density (ρ) and its Laplacian (∇^2^ρ) are two important parameters to quantify the interactions strength. The criterion for the existence of non-bonding interactions at the critical point is that the closed-shell interaction ρ is less than 0.1 a.u. and ∇^2^ρ possesses a small and positive value. The values of ρ and ∇^2^ρ at the bond critical points (BCPs) of CL-20/H_2_O_2_ range from 0.0161 to 0.0137 a.u. and 0.0454 to 0.0466 a.u., respectively, while those for CL-20/H_2_O from 0.0105 to 0.0121 a.u. and 0.0406 to 0.0489 a.u. It confirms that these bond critical points between O and H atoms represent the hydrogen bonds. The values of Eb of CL-20/H_2_O_2_ and CL-20/H_2_O are from 10.35 to 11.25 kJ mol^−1^ and 9.86 to 10.83 kJ mol^−1^, suggesting these hydrogen bond of between O and H atoms in CL-20/H_2_O_2_ are stronger than those in CL-20/H_2_O. The above RDF qualitative comparison combined with QTAIM quantitative topological analysis jointly verify the intrinsic difference in intermolecular interaction strength between the two solvates.

**Figure 5:**
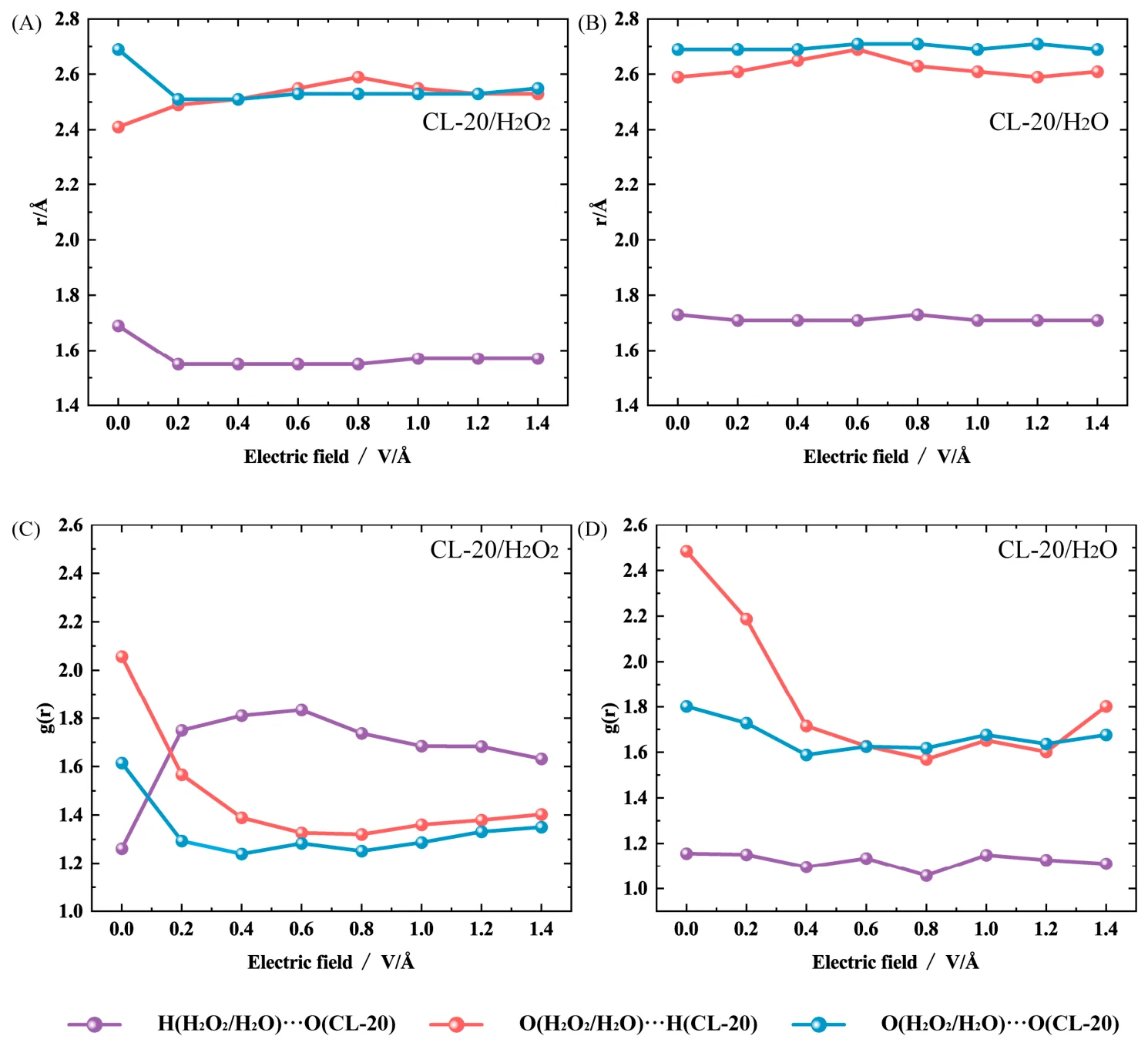
The variation of g(r) and r of CL-20/H_2_O_2_ (**A**,**C**) and CL-20/H_2_O (**B**,**D**) solvates with an external electric field applied along the *z*-axis.

Subsequently, the effect of external electric field strength on the intermolecular H···O and O···O atom pairs is discussed. Figure 5 presents the variations of g(*r*) and *r* of H···O and O···O interactions in the solvated complexes of CL-20/H_2_O_2_ and CL-20/H_2_O with the electric field strength. The distance between the intermolecular H···O atom pairs at 1.69 Å and the O···O interaction atoms at 2.69 Å in CL-20/H_2_O_2_ is shortened after applying an electric field, while the distance of atom pairs in the CL-20/H_2_O solvate remains almost unchanged, indicating that the intermolecular interactions between CL-20/H_2_O_2_ molecules are more sensitive to the influence of the electric field. In addition, the shortening of RDF gradually disappears at 0.4 V/Å, indicating that the electric field strength of 0.4 V/Å is the turning point for the structural changes of CL-20 based solvates, which are also more sensitive to CL-20/H_2_O_2_ solvates with electric field strength (Figure 5A,B).

### 2.3. Mean Square Displacement (MSD)

**Figure 6:**
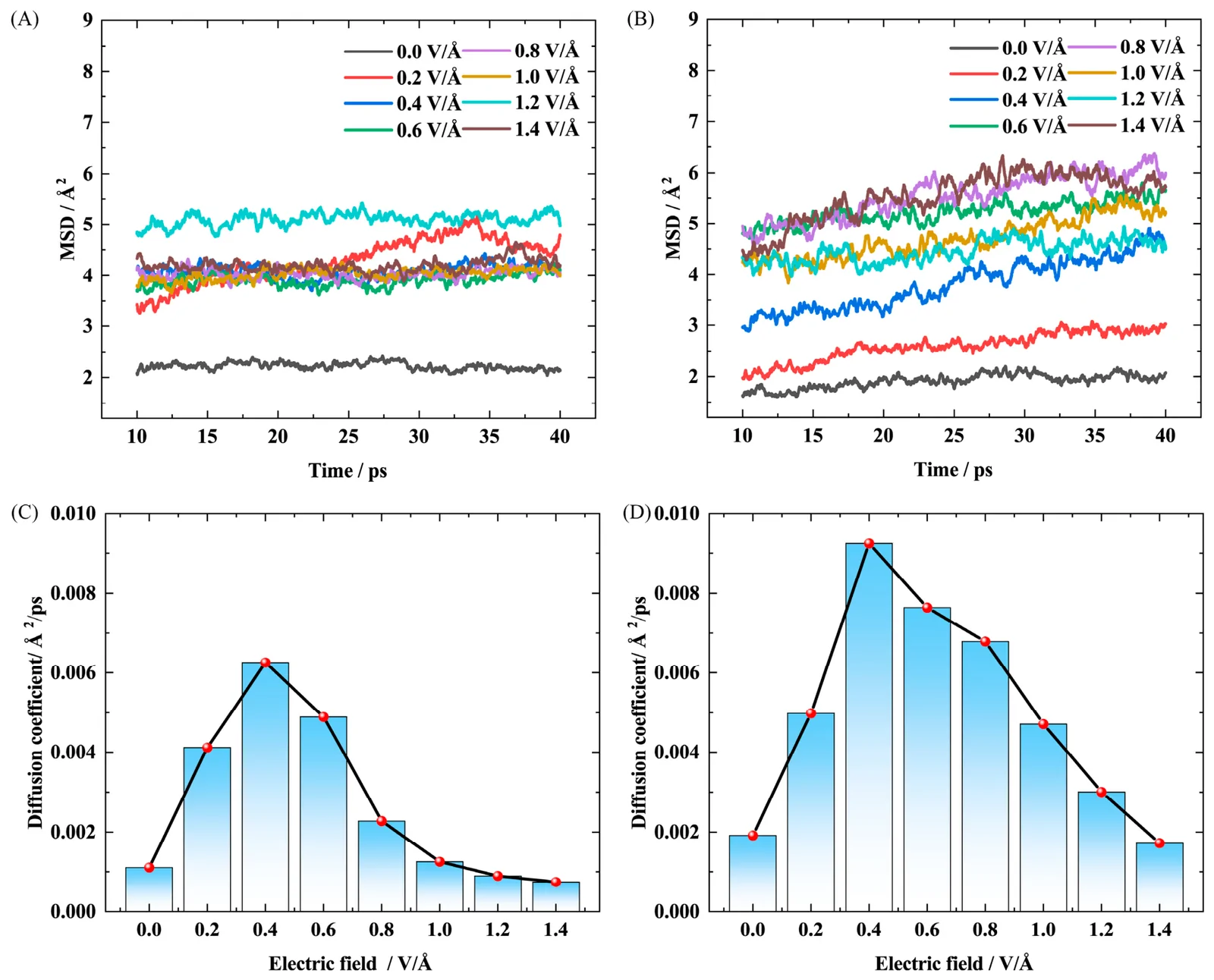
The mean square displacement and diffusion coefficient of CL-20/H_2_O_2_ (**A**,**C**) and CL-20/H_2_O (**B**,**D**) solvates with an external electric field applied along the *z*-axis.

The mean-square displacement (MSD) and diffusion coefficient (D) are calculated by analyzing molecular dynamic trajectories under applied electric fields, as illustrated in Figure 6. Figure 6A,B reveal that the H_2_O molecule inside the CL-20-based energetic solvate exhibits a relatively larger MSD value. The diffusion coefficient fluctuates remarkably with electric field intensity, showing an upward trend followed by a decline and peaking at 0.4 V/Å. CL-20/H_2_O and CL-20/H_2_O_2_ share identical variation tendency of diffusion coefficient with the maximum value obtained at 0.4 V/Å, demonstrating that the intermolecular interaction between CL-20 and solvent molecules undergoes obvious transition at this electric field. The larger diffusion coefficient indicates faster diffusion of molecules or atoms. Compared with the CL-20/H_2_O_2_ solvates, CL-20/H_2_O is more susceptible to the influence of field strength changes. Moreover, the H_2_O molecules in α-CL-20 possess a higher diffusion rate than H_2_O_2_ molecules. This further indicates that the interaction between CL-20 and H_2_O_2_ molecules is stronger, and the stability of the crystal lattice is enhanced.

### 2.4. The Interaction Energy of the Trigger Bond

Based on the hot-spot theory, the trigger bond is the weakest chemical bond in energetic materials and the most likely to break when exposed to external stimuli, thereby causing the explosive to decompose or explode. Here, the maximum length and the interaction energy of the trigger bond (denoted as *E*_N-N_ in the N-NO_2_ bond) were selected in CL-20-based energetic solvates as practical indicators for predicting explosive sensitivity. The statistical distribution of trigger bond lengths offers fundamental insights into the stability and safety of explosives. A lower *E*_N-N_ correlates with a weaker trigger bond, facilitating the initiation of decomposition or detonation. The expression of *E*_N-N_ is as follows:

(1) $$E_{N-N}=\frac{E_{1}-E_{2}}{n}$$

where *E*_1_ represents the total energy of the system in its thermodynamic equilibrium state and *E*_2_ is the total energy of the system when it reaches equilibrium after fixing the three-dimensional coordinates of all N atoms in CL-20. For the CL-20, All N atoms constitute the cage skeleton of CL-20 and participate in forming N-NO_2_ trigger bonds. By comparing *E*_1_ and *E*_2_, we can obtain the intrinsic interaction energy of the N-NO_2_ trigger bond, and this calculation strategy can effectively eliminate the energy perturbations originating from lattice vibration and structural deformation. *n* is the number of N-NO_2_ bonds in the cocrystal system.

**Figure 7:**
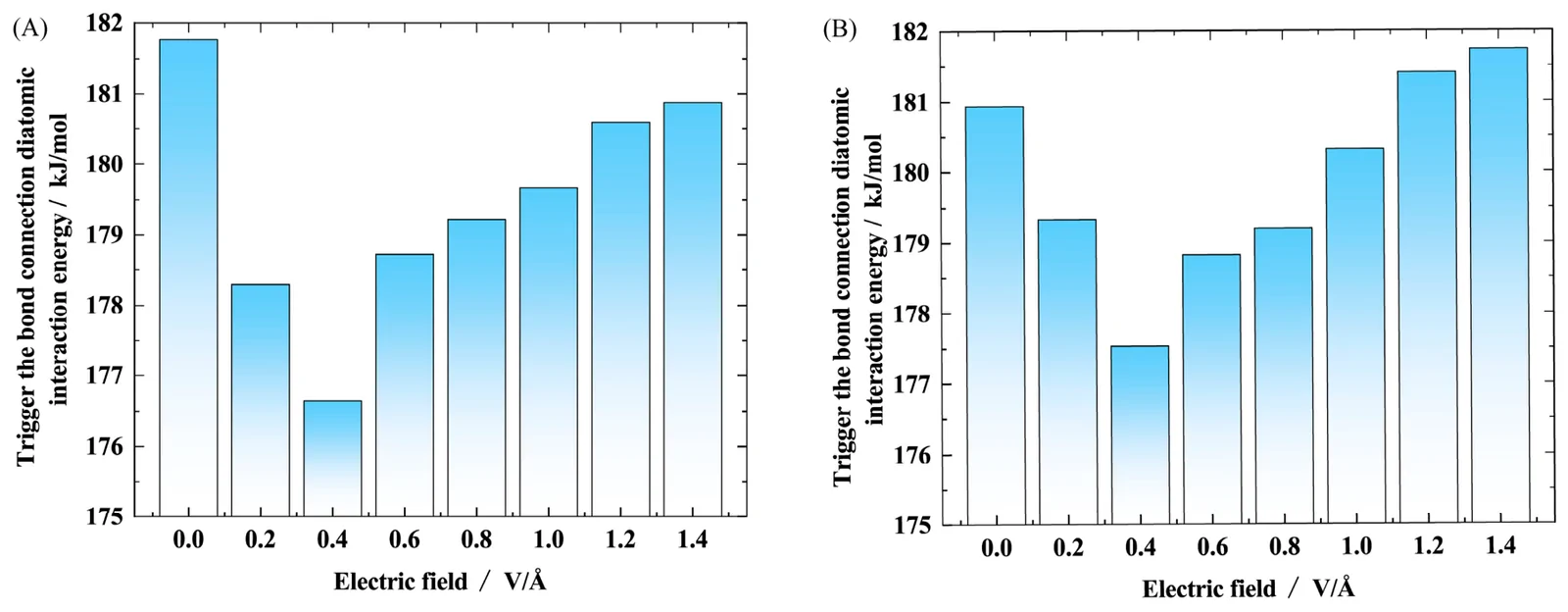
The interaction energy of the trigger bond (*E*_N-N_) for CL-20/H_2_O_2_ (**A**) and CL-20/H_2_O (**B**) solvates with an external electric field applied along the *z*-axis.

The interaction energy of the trigger bond (*E*_N-N_) for is shown in Figure 7. Notably, *E*_N-N_ of the trigger bond N-NO_2_ shows a trend of initial decrease and subsequent gradual increase with the increase of the EEF. Under no electric field, the value of *E*_N-N_ of CL-20/H_2_O_2_ and CL-20/H_2_O are 182.58 kJ/mol and 180.95 kJ/mol, respectively. Under the EEF at 0.4 V/Å, the *E*_N-N_ decreases to varying extents and reaches the minimum, where CL-20/H_2_O_2_ and CL-20/H_2_O are respectively at 176.51 kJ/mol and 177.45 kJ/mol. Further, CL-20/H_2_O_2_ exhibits a larger reduction of *E*_N-N_, which demonstrates that H_2_O_2_ molecules amplify the structural perturbation of the EEF on CL-20 and render the solvate more sensitive under electric field environment. As the electric field rises from 0.4 to 1.4 V/Å, the interaction energy of the trigger bond gradually rebounds. This behavior is attributed to the enhanced molecular polarity and lattice contraction, which restrict the structural deformation and dissociation of the N-NO_2_ trigger bond and moderately improve bond stability.

Nevertheless, the *E*_N-N_ values under the EEF are all lower than the zero-field system overall, the positive *z*-direction electric field weakens the N-NO_2_ trigger bond and the *E*_N-N_, and the molecular sensitivity increases. The reduction of *E*_N-N_ of the trigger bond N-NO_2_ originates from the directional perturbation of electric fields on the intermolecular hydrogen bond network and van der Waals interactions, which irreversibly disturbs the molecular stacking and dipole arrangement of CL-20 and guest molecules.

This is consistent with previous reports on the electric field response of CL-20/H_2_O_2_ solvates [38], where H_2_O_2_ accelerates the transformation of the CL-20 skeleton structure and weakens adjacent C-N chemical bonds due to the swinging of nitro groups, thereby reducing the stability of CL-20 and improving system sensitivity. This can be explained by the fact that H_2_O_2_ and the hydroxyl radicals (OH·) generated from its decomposition promote the destruction of the cage-like skeleton of CL-20 by reactive force field molecular dynamics simulations (ReaxFF RMD simulation), while generating a large number of water molecules and releasing heat. Therefore, the decomposition rate of CL-20/H_2_O_2_ is higher than that of ε-CL-20.

**Figure 8:**
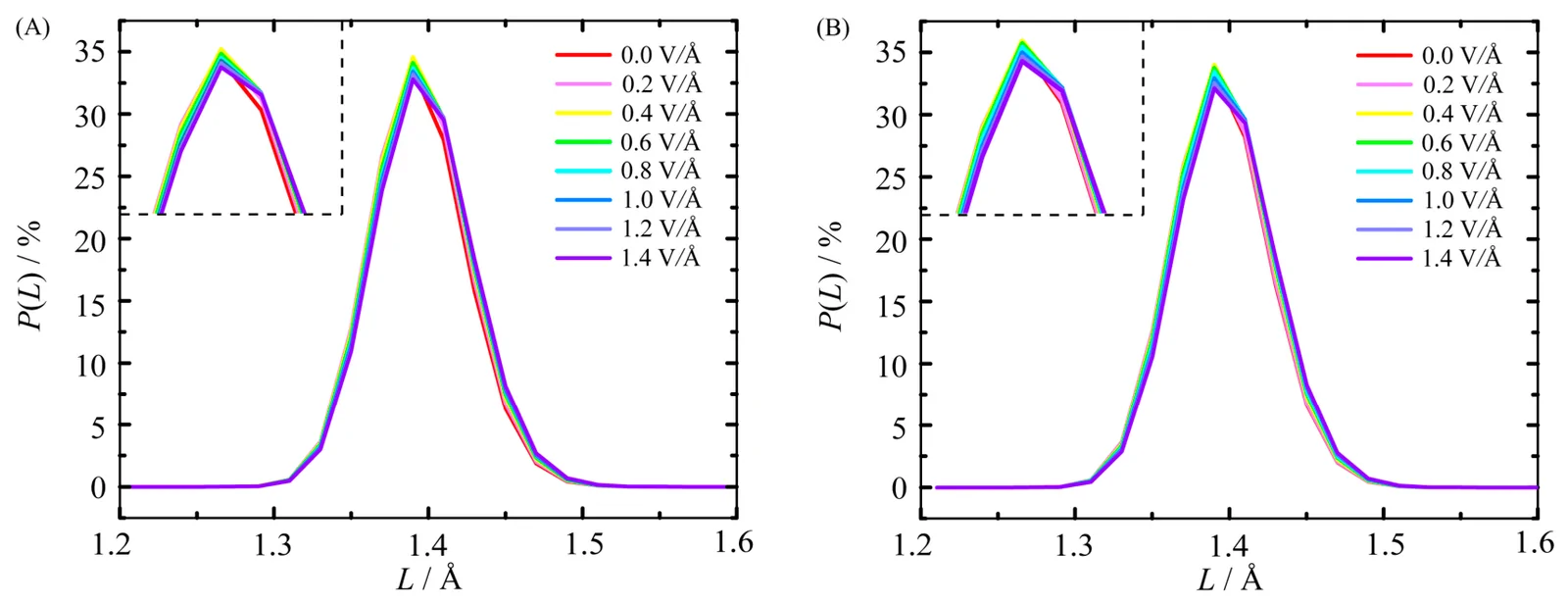
The trigger bond length distribution of CL-20/H_2_O_2_ (**A**) and CL-20/H_2_O (**B**) solvates.

Figure 8 displays the distributions of bond lengths of the N-NO_2_ trigger bond of CL-20/H_2_O_2_ and CL-20/H_2_O solvates in the equilibrium system obtained from MD simulation. The bond length distribution presents an approximately symmetrical Gaussian distribution, suggesting that their most probable bond length (*L*_prob_) is consistent with the average bond length (*L*_ave_). The bond length of N-NO_2_ in CL-20/H_2_O_2_ and CL-20/H_2_O is 1.39 Å in the absence of an external electric field, which is close to the experimental value (the bond length of N-NO_2_ in ε-CL-20 is 0.138 nm) [25].

With the increase of positive z-direction external electric field strength, the distribution of N-NO_2_ bond lengths in all crystals gradually narrows and shifts towards the shorter bond lengths. This indicates obvious geometric contraction of the trigger bond under electrostatic polarization. Notably, significant structural variation occurs at 0.4 V/Å, which corresponds to the critical conformational transition point of the CL-20 solvate systems.

### 2.5. Cohesive Energy Density

**Figure 9:**
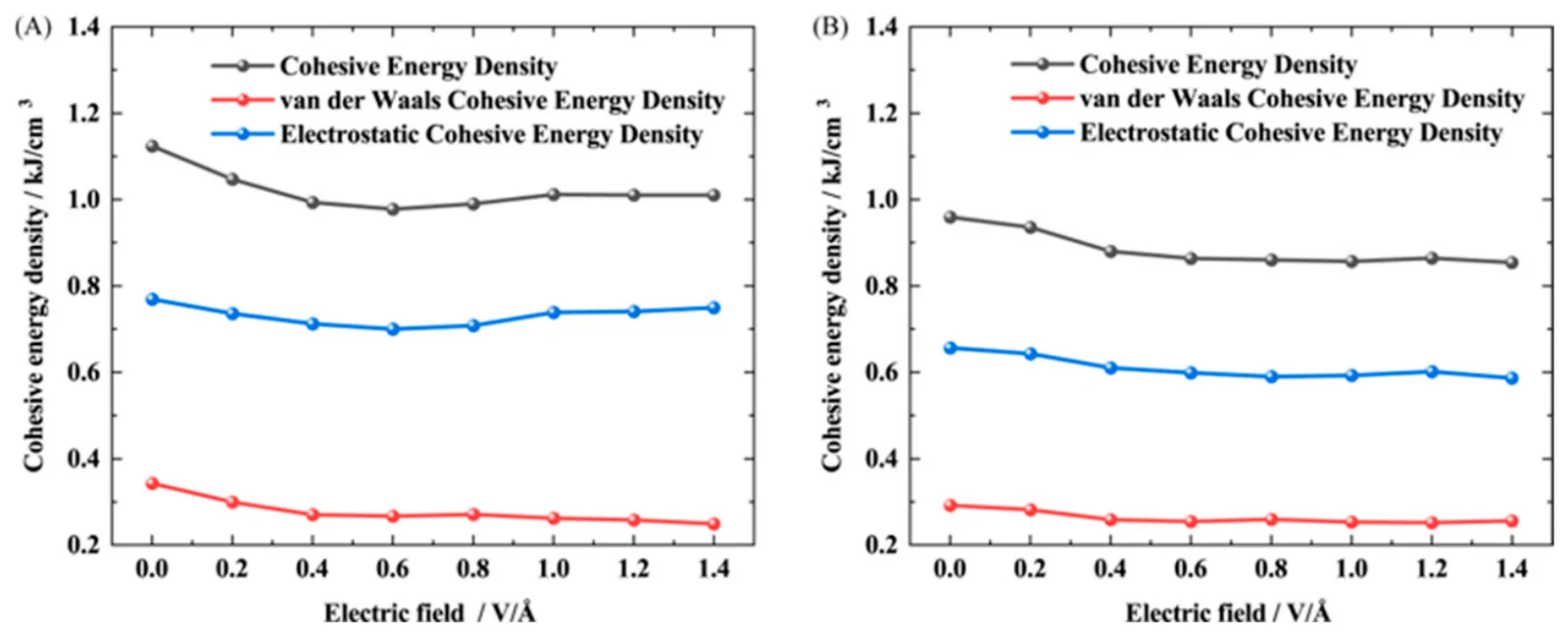
Cohesive energy density (CED) of CL-20/H_2_O_2_ (**A**), CL-20/H_2_O (**B**) solvates with an external electric field applied along the *z*-axis.

Cohesive energy density (CED) quantitatively characterizes the strength of the intermolecular interactions within a crystal. Figure 9 presents the cohesive energy density of the CL-20/H_2_O_2_ and CL-20/H_2_O solvates at 0.0 V/Å to 1.4 V/Å, together with the separate contributions from van der Waals forces and electrostatic forces. Under different electric field intensities, the cohesive energy density of CL-20/H_2_O_2_ is consistently greater than that of CL-20/H_2_O. When the electric field intensity is below 0.4 V/Å, the CED decreases as the electric field intensity increases. When the electric field intensity exceeds 0.4 V/Å, the cohesive energy density of CL-20/H_2_O_2_ increases and stabilizes, and is predominantly governed by electrostatic interactions. In contrast, the CED of CL-20/H_2_O decreases continuously as the electric field intensity increases, which mainly originates from the weakening of electrostatic forces. This further indicates that hydrogen bonding is the primary intermolecular driving force in CL-20-based solvates, and that the interaction between CL-20 and H_2_O_2_ molecules is more stable.

Although the external electric field compresses the N-NO_2_ bond geometry, the reduced trigger bond interaction energy under all applied electric fields and the continuous decline in cohesive energy density demonstrate that the positive EEF generally weakens both intramolecular trigger bonding and intermolecular interactions. Accordingly, the geometric contraction of the N-NO_2_ bond cannot independently improve the overall stability of CL-20-based energetic solvates. Therefore, it also reflects that the trigger bond shortening is merely a geometric feature rather than reliable evidence of improved structural stability.

### 2.6. Mechanical Properties

**Figure 10:**
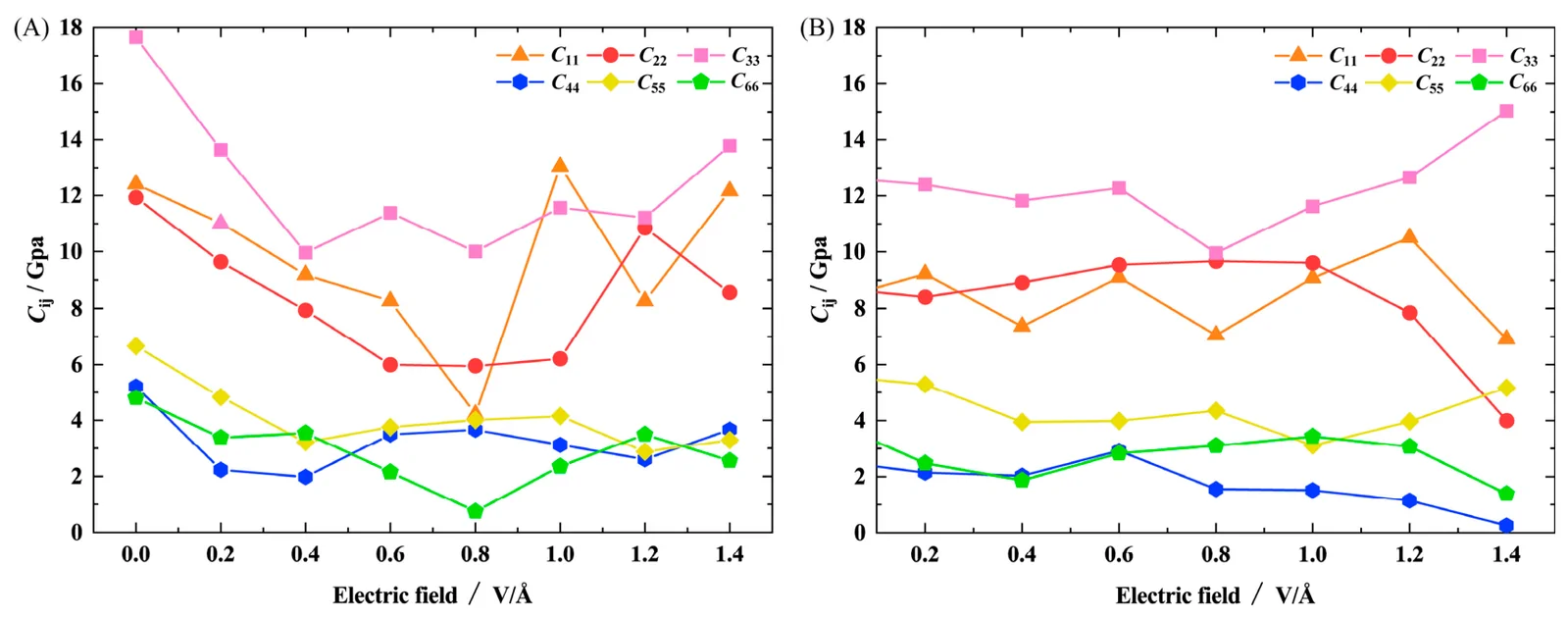
The elastic coefficients of CL-20/H_2_O_2_ (**A**) and CL-20/H_2_O (**B**) solvates under different external electric fields applied along the *z*-axis.

Mechanical properties are one of the most critical indicators of energetic materials characterization [22]. Three mechanical parameters including elastic stiffness constants, engineering moduli and Poisson’s ratio were calculated, with elastic constants describing the intrinsic stress–strain relationship of the crystalline materials. Figure 10 summarizes the evolution of the elastic constants of CL-20/H_2_O_2_ and CL-20/H_2_O solvates under the external electric field. The diagonal elements of the elastic constant tensor exhibit obvious anisotropic mechanical responses in CL-20-based solvates. Comparing the elastic constants C11, C22, and C33 at 298 K and 101 kPa, CL-20/H_2_O_2_ solvate possesses superior shear thermal stability. The elastic constants of CL-20/H_2_O_2_ solvates increase continuously with the electric field loading apart from along the b-axis.

For CL-20/H_2_O, elastic constants remain stable at a low electric field and fluctuate markedly at high field strength. The elastic coefficient of the *a*- and *b*-axis decreases but that of the c-axis increases with the EEF. This indicates that CL-20/H_2_O solvates are more sensitive to external electric-field regulation and can be used as an effective means to reduce their elastic constants. This is also related to the stronger intermolecular hydrogen bonding in CL-20/H_2_O_2_, which stabilizes the crystal structure. Once the external electric field exceeds the critical value at the conformational transition point, the variation amplitude of elastic coefficients is significantly enlarged, which confirms the regulatory effect of the external electric field on the structure of CL-20-based solvated species.

Engineering moduli are used to characterize the rigidity and elastic deformation resistance of energetic crystals. Young’s modulus (*E*) and shear modulus (*G*) directly reflect structural rigidity, with larger values corresponding to stiffer crystals. Bulk modulus (*B*) is adopted to evaluate compressive fracture resistance, calculated by averaging the Voigt and Reuss bounding values; a higher bulk modulus indicates stronger anti-fracture capacity. Young’s modulus *E* and Poisson’s ratio *γ* can be derived from bulk modulus *B* and shear modulus *G* via the following formulas [39]:

(2) E=2G(1+γ)=2B(1−2γ)

(3) $$\gamma =\frac{3B-2G}{2(3B+G)}$$

**Figure 11:**
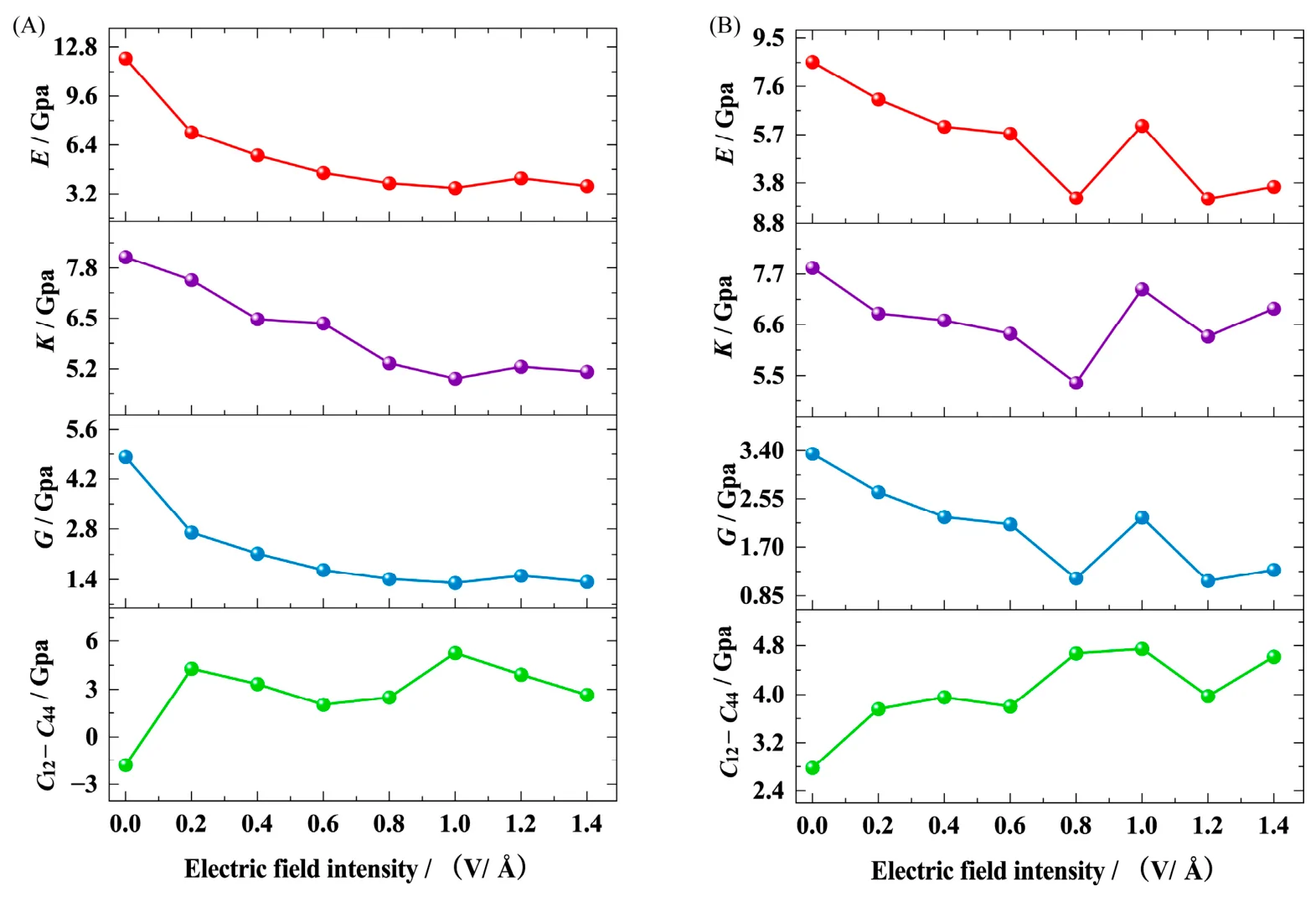
Mechanical performance parameters of CL-20/H_2_O_2_ (**A**) and CL-20/H_2_O (**B**) solvates under different external electric fields applied along the *z*-axis.

Table 1 and Table 2 list the Young’s modulus *E*, bulk modulus *B*, shear modulus *G*, Poisson’s ratio *γ*, and *B*/*G* of CL-20/H_2_O_2_ and CL-20/H_2_O solvates under different external electric field. Figure 11 presents mechanical performance parameters of CL-20/H_2_O_2_ and CL-20/H_2_O solvates under different external electric field applied along the *z*-axis. Relative to the zero-field state, *E*, *B*, and *G* of the two CL-20-based solvates show an overall decrease under the electric field, except at 1.0 V/Å for CL-20/H_2_O solvates. This demonstrates that *z*-axis directional electric field weakens the overall structural rigidity of CL-20 energetic solvates. Furthermore, the modulus fluctuation range of CL-20/H_2_O is far larger than that of CL-20/H_2_O_2_, indicating the rigidity of CL-20/H_2_O is more susceptible to electric field disturbance.

Poisson’s ratio (*γ*) is a typical indicator for crystal plastic deformation, with a typical range of 0.2–0.4 for condensed crystals. External electric field markedly elevates the Poisson’s ratio of both solvates. As listed in Table 1 and Table 2, the Poisson’s ratio of CL-20/H_2_O_2_ rises from 0.25 to 0.39, while that of CL-20/H_2_O increases from 0.28 to 0.31. The elevated Poisson’s ratio reveals enhanced plastic deformation capacity and larger elastic elongation under electric field loading. The *B/G* ratio is another ductility-brittleness discriminant: a higher *B/G* value corresponds to better ductility. The *B/G* values of both CL-20 solvates increase significantly under electric field excitation. In conclusion, the external electric field effectively improves the plastic ductility of energetic solvate crystals.

Cauchy pressure (*C*_12_–*C*_44_) is widely used to judge the ductility or brittleness of energetic crystals: positive Cauchy pressure represents ductile behavior, whereas negative value means brittle characteristic. The Cauchy pressure of both crystals increases monotonically with rising electric field intensity. At ambient conditions (298 K, 101 kPa) without electric field, CL-20/H_2_O_2_ behaves as brittle with negative Cauchy pressure, while pristine CL-20/H_2_O shows intrinsic ductility. The ductility of both solvate crystals is greatly strengthened under the electric field. This result confirms that the external electric field suppresses crystal brittleness and improves ductility efficiently.

## 3. Materials and Methods

The crystal structures of CL-20/H_2_O_2_ and CL-20/H_2_O solvates were obtained by X-ray diffraction from the Cambridge Crystallographic Database v6 (CCDC, Cambridge, UK). The unit cells of CL-20/H_2_O_2_ and CL-20/H_2_O solvates are enlarged along 4a × 4b × 2c three directions to gain the supercells of CL-20/H_2_O_2_ (5120 atoms) and 4a × 4b × 2c CL-20/H_2_O (4992 atoms).

In addition, some other studies [24,25] have shown that the EEF has a significant effect on the trigger bond when it is parallel to the trigger bond. In this work, the structural optimization and Laplacian bond order (LBO) analysis were performed at the 6-311G++(d, p) basis set level using the B3LYP method [40], and wave function information was calculated using the Gaussian 09 program [41], imported into Multiwfn 3.8 [42,43] and VMD v1.9.3 [44] was used for analysis. According to the result of Laplacian bond order calculation (Figure S1), the N-NO_2_ bond was selected as the trigger bond. Among six spatially distinct N-NO_2_ bonds of the CL-20 cage, the trigger bond with the lowest bond order was selected as the characteristic bond. VMD was adopted to rotate the crystal configuration to align this specific N-NO_2_ bond parallel to the *z*-axis, and the positive z-direction was defined as the positive direction of the applied electric field. The same orientation strategy was applied to the CL-20/H_2_O and CL-20/H_2_O_2_ solvates for consistent comparison. The external electric field was applied along the *z*-axis with a range from 0.0 to 1.4 V·Å^−1^, with an interval of 0.2 V·Å^−1^.

Geometry optimization was performed to minimize interatomic contacts using the COMPASSII force field, which has been validated for ammonium nitrate energetic materials containing H, O, and N atoms [45]. Then, the optimized structures were equilibrated under the NPT ensemble at 298 K and 0 GPa for a total of 200 ps with 0.1 fs time steps, and the system was sufficiently relaxed, as indicated by the fluctuation profiles of temperature and energy. Then, MD simulations were performed under the NPT ensemble with a total simulation time of 500 ps and a time step of 0.1 fs. The Berendsen thermostat and barostat were employed to maintain the temperature and pressure at 298 K and 0 GPa, respectively, with decay constants of 0.1 ps for both. Van der Waals interactions were computed using the atom-based summation method with a long-range correction and a cutoff distance of 15.5 Å. Electrostatic interactions were evaluated using the Ewald summation method with a buffer width of 0.5 Å and an accuracy of 0.001. The mean-squared displacement (MSD) is analyzed for the first 300 ps of motion trajectories of solvent molecules in energetic solvates, and the diffusion coefficient (*D*) is then calculated from the resulting MSD [46]. All MD simulations under external electric fields were conducted using the Forcite Module and Perl scripts in Materials Studio 8.0.

## 4. Conclusions

In this work, theoretical simulations were conducted to explore the structural evolution, intermolecular interaction, trigger bond stability, sensitivity and mechanical properties of CL-20/H_2_O_2_ and CL-20/H_2_O solvates under a *z*-axis external electric field (EEF). Compared with CL-20/H_2_O, CL-20/H_2_O_2_ solvates possess stronger intermolecular O-H···O/C-H···O hydrogen bonds. The hydrogen bond networks can buffer the variation of cell parameters under external electric field, suppress molecular diffusion and decrease the interaction energy of the trigger bond of N-NO_2_ (*E*_N-N_) and cohesive energy density (CED). The conformers of CL-20 molecules transform from the α-phase to the thermodynamically stable ε phase after 0.4 V·Å^−1^, suggesting that the orientation of the NO_2_ groups is adjustable and that the phase transition occurs under the applied field. The reduced stiffness and enhanced lattice ductility of mechanical properties enables effective buffering and uniform dispersion of external stress through conformational rearrangement of CL-20 molecules and flexible solvation interactions. This suppresses hotspot generation and improves the thermal safety of energetic crystals under impact loading. This work reveals the electric response mechanism at the molecular level of CL-20 solvates and provides theoretical guidance for electric-field modification.

## Figures and Tables

**Table 1:** Mechanical properties of CL-20/H_2_O_2_ solvates under different electric fields.

Modulus	0.0 V/Å	0.2 V/Å	0.4 V/Å	0.6 V/Å	0.8 V/Å	1.0 V/Å	1.2 V/Å	1.4 V/Å
*E*	12.06	7.24	5.73	4.59	3.90	3.59	4.23	3.72
*B*	8.09	7.49	6.47	6.37	5.34	4.95	5.25	5.12
*G*	4.82	2.70	2.12	1.67	1.41	1.30	1.51	1.33
*K/G*	1.68	2.77	3.06	3.83	3.78	3.81	3.48	3.84
*γ*	0.25	0.34	0.35	0.38	0.38	0.38	0.40	0.39
*C*_12_–*C*_44_	−1.78	4.29	3.33	2.03	2.49	5.26	3.92	2.65

**Table 2:** Mechanical properties of CL-20/H_2_O solvates under different electric fields.

Modulus	0.0 V/Å	0.2 V/Å	0.4 V/Å	0.6 V/Å	0.8 V/Å	1.0 V/Å	1.2 V/Å	1.4 V/Å
*E*	7.84	6.84	6.69	6.41	5.34	7.37	6.34	6.95
*B*	6.09	6.40	6.57	7.32	5.34	4.95	7.06	5.86
*G*	3.34	2.67	2.23	2.10	1.16	2.22	1.11	1.31
*B/G*	1.82	2.40	2.95	3.48	4.62	2.23	6.36	4.49
*γ*	0.29	0.32	0.37	0.36	0.30	0.30	0.36	0.34
*C*_12_–*C*_44_	2.78	3.76	3.96	3.80	4.68	4.75	3.98	4.62

## Data Availability

The original contributions presented in this study are included in the article. Further inquiries can be directed to the corresponding author.

## Supplementary Materials

The following supporting information can be downloaded at: https://www.mdpi.com/article/10.3390/ijms27167209/s1.
