# Supplementary material for: Physical Responses to the Structural Evolution of CL-20-Based Energetic Solvates with H2O2 and H2O Under External Electric Field
Source: Int J Mol Sci. 2026 Aug 12;27(16):7209. doi: 10.3390/ijms27167209 (PMC13513717; doi:10.3390/ijms27167209)
Supplement: Supplementary file 1 [file ijms-27-07209-s001.zip › ijms-4453193-supplementary.pdf]

Supporting Information

Physical Responses to the Structural Evolution of CL-20-based energetic solvates with H_2O_2 and H_2O under External Electric Field

Lu Shi [1*], Li Fan [1], Huaya Sun [1], Daichuan Ma [2]

[1] College of Chemistry and Materials Engineering, Mianyang Teachers' College, Mianyang, Sichuan 621000, China; 18280769523@163.com (L. F); shy1738082@outlook.com (H. S)

[2] Analytical & Testing Center, Sichuan University, Chengdu, Sichuan 610064, China; dcma@scu.edu.cn

* Corresponding Author Emails: shilulu0224@163.com

1. Bond order

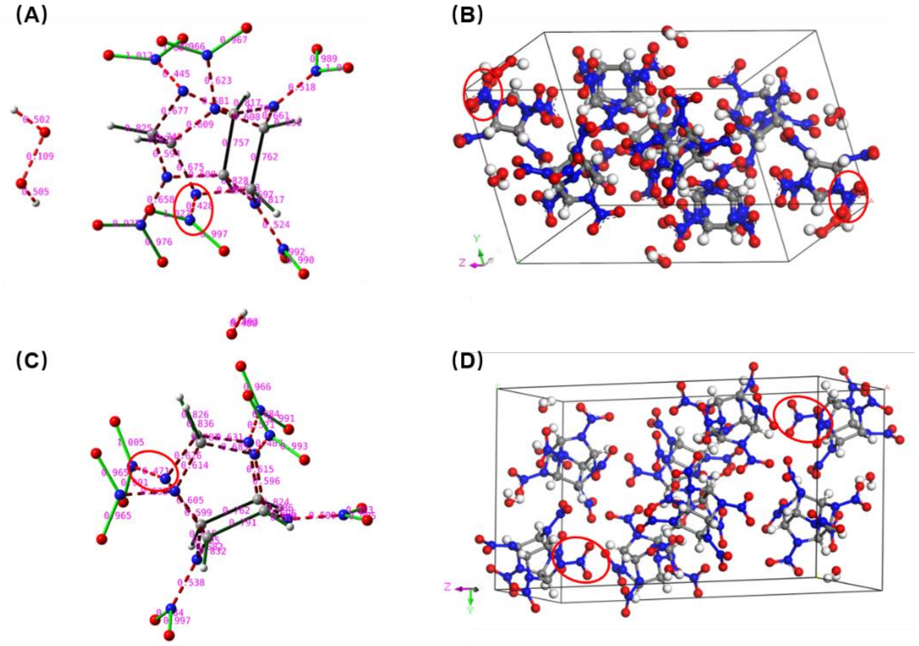

Figure S1 Laplacian Bond order of CL-20 molecules in the CL-20/H_2O_2 (A) and CL-20/H_2O (C) solvates and the orientation of N-NO_2 bond in the CL-20/H_2O_2 (B) and CL-20/H_2O (D). The N-NO_2 groups with respect to z-axis marked in red circles.

In general, the trigger bond in energetic molecules is weak and easy to break, which is associated with low bond order, the smaller the electron cloud density, and weak orbital overlap between bonded atoms. The Laplacian bond order (LBO) analysis is performed at the 6-311G++(d, p) basis set level using the B3LYP method. Then, we selected a characteristic N-NO_2 trigger bond from the six N-NO_2 bonds of the CL-20 cage in the CL-20/H_2O_2 solvate; this selected bond exhibits the lowest bond order (Figure S1A and C). Subsequently, VMD software was utilized to orient the crystal such that this characteristic trigger bond is parallel to the z-axis, and the positive z-direction was defined as the positive direction of the applied external electric field (Figures S1B and D). An identical orientation setup was adopted for the CL-20/H_2O solvate to ensure comparative consistency.

2. AIM analysis

Based on QTAIM (Quantum Theory of Atoms in Molecules) [1-2], the more detailed information about the hydrogen-bonding interactions can be obtained by conducting topological analyses on the molecular clusters extracted from the CL-20/H_2O_2 and CL-20/H_2O solvates (Figure S2). At the critical points (CPs) of the particular clusters, we can acquire the kinetic energy density (G_b), the potential energy density (V_r), and the hydrogen-bonding energy (E_b), which can characterize the types and strength of the hydrogen bonding. The expressions are presented as follows:

$$G_b=(3/10)\left(3\pi^2\right)^{2/3}\rho^{5/3}+(1/6)\nabla^2\rho$$

$$V_b=(\hbar/4m)\left(3\pi^2\right)\nabla^2\rho-2G_b$$

$$E_b=1/2V_b$$

where ρ denotes the electron density and $\nabla^2\rho$ signifies the Laplacian. These calculations were carried out at the B3LYP/ 6-311G(d,p) level using the Gaussian 09 package, and the subsequent analysis was performed using Multiwfn 3.8.

According to the clusters constructed above using the atoms in molecules (AIM) theory, hydrogen-bonding energies can be described accurately based on the potential energy densities of the (3,1) critical point. Tables S1 present the topological parameters of the hydrogen bonds at the (3,1) bond critical point in the CL-20/H_2O_2 and CL-20/H_2O solvates, respectively. The electron density (ρ) and its Laplacian ($\nabla^2\rho$) are two important parameters to quantify the

interactions strength. The criterion for the existence of non-bonding interactions at the critical point is that the closed-shell interaction ϱ is less than 0.1 a.u. and $\nabla^2\varrho$ possesses a small and positive value. The values of ϱ and $\nabla^2\varrho$ at the bond critical points (BCPs) of CL-20/H_2O_2 from 0.0161 to 0.0137 a.u. and 0.0454 to 0.0466 a.u., respectively, while those for CL-20/H_2O from 0.0105 to 0.0121 a.u. and 0.0406 to 0.0489 a.u. It confirms that these bond critical points between O and H atoms represent the hydrogen bonds. The values of Eb of CL-20/H_2O_2 and CL-20/H_2O are from 10.35 to 11.25 kJ mol-1 and 9.86 to 10.83 kJ mol^{-1}, suggesting these hydrogen bond of between O and H atoms in CL-20/H_2O_2 are stronger than those in CL-20/H_2O.

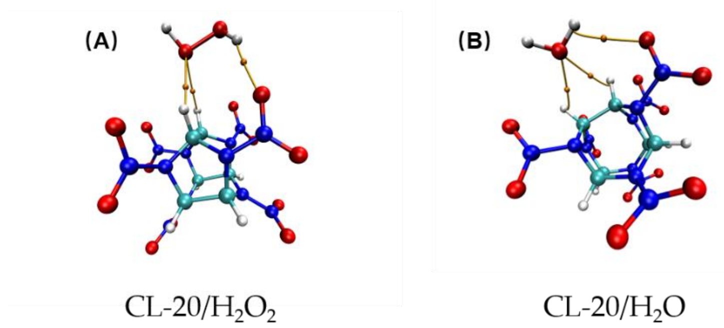

Figure S2 Topological analysis of hydrogen bonds in CL-20/H_2O_2 (A) and CL-20/H_2O (B) solvates at the (3, -1) critical point

Table S1 Topological parameters of hydrogen bonds in CL-20/H_2O_2 and CL-20/H_2O solvates at the (3, -1) critical point: hydrogen bond length (Å), angle (1), density of all electrons (ϱ, e Å^{-3}), Laplacian of electron density ($\nabla^2\varrho$, e Å^{-5}), potential energy density (V_b), and hydrogen bond energy (E_b, kJ mol^{-1})

Solvates	Interactions	D-H	H···A	D-H···A	ϱ × 100	$\nabla^2\varrho$ × 10	V_b × 100	E_b
CL-	C-H···O	0.9671	2.4508	123.1233	1.2178	0.4576	-0.7872	10.35
20/H_2O_2	C-H···O	0.9671	2.2826	145.2117	1.3785	0.4658	-0.8441	11.08
	O-H···O	0.9699	2.1297	153.2779	1.1611	0.4542	-0.8572	11.25
CL-	C-H···O	1.0812	2.4412	104.8181	1.1296	0.4624	-0.7812	10.25
20/H_2O	C-H···O	1.0803	2.4147	105.8805	1.2093	0.4891	-0.8251	10.83
	O-H···O	0.9636	2.3784	116.7505	1.0524	0.4064	-0.7514	9.86

References

1. Bader, R.F.W. A Quantum Theory of Molecular Structure and Its Applications. *Chem. Rev.* ***1991***, *91*(5), 893-928. DOI:10.1002/chin.199203324.
2. Espinosa, E.; Molins, E.; Lecomte, C. The Hydrogen Bond Strength in Terms of the Topological Properties of the Electron Density Distribution. *Chem. Phys. Lett.* ***1998***, *285*(3), 170-173. DOI:10.1016/S0009-2614(98)00036-0.
